# P-1274. Mpox: Characterization and Clinical Outcomes of Patients Attended in Health Service Providing Institutions in Colombia

**DOI:** 10.1093/ofid/ofae631.1455

**Published:** 2025-01-29

**Authors:** Carlos A Alvarez-Moreno, María Lucia Mesa Rubio, Leonardo Arévalo Mora, Julián Felipe Porras Villamil, Ludovic Reveiz, Juan Carlos Alzate, Antoine Chaillon, Ilich de la Hoz, Kurbonov Firdavs, Jamie Rylance, Sandra Valderrama, Claudia Aristizabal, Otto A Sussmann, Javier Andrade, Carolina Murillo, Ximena Galindo, Daniela Martínez, Lisandra Arango, Ernesto Martínez Buitrago, Karyna Reyes, Mónica Mantilla Suárez, Juana Gutierrez, Alexandra Garay, Wendy Pinzón, Ana Crispin

**Affiliations:** Clínica Colsanitas, Clínica Universitaria Colombia, Bogotá, Distrito Capital de Bogota, Colombia; PAHO, Colombia, Bogota, Distrito Capital de Bogota, Colombia; CEPAIN Bogotá, Bogotá DC, Cundinamarca, Colombia; PAHO, Colombia, Bogota, Distrito Capital de Bogota, Colombia; PAHO, Washington, Washington, Washington; Instituto de Evaluación Tecnológica en Salud (IETS), Bogota, Distrito Capital de Bogota, Colombia; World Health Organization, Washington, Washington; World Health Organization, Washington, Washington; World Health Organization, Washington, Washington; World Health Organization,, Washington, District of Columbia; Hospital Universitario San Ignacio, Pontificia Universidad Javeriana, Bogotá, Colombia, Bogota, Distrito Capital de Bogota, Colombia; Unisanitas, Bogota, Distrito Capital de Bogota, Colombia; Asistencia Científica de Alta Complejidad, Infectoclinicos, Bogota, Cundinamarca, Colombia; Infectoclinicos, Bogota, Distrito Capital de Bogota, Colombia; Hospital La María, Bogota, Distrito Capital de Bogota, Colombia; Corporación de Lucha contra el Sida, Cali, Valle del Cauca, Colombia; Hospital Alma Mater de Antioquia, Bogota, Distrito Capital de Bogota, Colombia; Hospital Alma Mater de Antioquia, Bogota, Distrito Capital de Bogota, Colombia; Universidad del Valle, Director Grupo VIHCOL, Cali, Valle del Cauca, Colombia; Hospital Universitario del Valle, Bogota, Distrito Capital de Bogota, Colombia; CEPAIN, Bogotá, Distrito Capital de Bogota, Colombia; Virrey Solís IPS, Bogota, Distrito Capital de Bogota, Colombia; Clínica de La Sabana, Bogota, Distrito Capital de Bogota, Colombia; Clínica de La Sabana, Bogota, Distrito Capital de Bogota, Colombia; Clínica de La Sabana, Bogota, Distrito Capital de Bogota, Colombia

## Abstract

**Background:**

The monkeypox virus, part of the Orthopoxvirus genus, triggered a worldwide outbreak in 2022. While this outbreak had widespread effects, there's limited information on mpox's specific impact in Colombia, particularly in terms of how it is managed, its burden, and its epidemiology. This research seeks to examine the medical context, clinical variations, and health consequences in individuals diagnosed with mpox, especially those with HIV in Colombian health institutions

**Methods:**

This retrospective study was conducted in health institutions in Colombia based on clinical records from Jan 2022 to Dec 2023. . Participants in the study were diagnosed through molecular methods and their clinical evolution was tracked through medical records. CD4 groups, BMI groups, viral suppression status, and HIV status were used to analyse the results.

**Results:**

One thousand four hundred thirteen (1,413, 97.2% male) individuals, including 2.6% identified as healthcare workers were included in this study. Concomitant sexually transmitted diseases were common, affecting 30.1%, mainly syphilis (80%) and *Neisseria gonorrhoeae* (16.4%). 54% of the population (764/1413 individuals, 99.3% male) were persons living with HIV (PWH), and almost one-third (31%, n=284) of participants had concomitant sexually transmitted diseases and HIV

PWH also had a higher proportion of gastrointestinal symptoms and genital symptoms. An important difference between HIV-positive and negative patients is that the former had a higher proportion of syphilis (p-value 0.5909) and other STIs (p-value 0.0026). Although PWH were younger (34.3±7.7 vs. 37.7±7.5), the difference in mean age was not statistically significant, although the differences in proportion were (15 to 44 years group, p-value 0.0097).

**Conclusion:**

The evidence presented shows that half of the population were people living with HIV, and the presence of mpox was not statistically significant in people with unsuppressed viral load or with low CD4 levels. The evidence provided by this study, whose results were standardized by the CRFs, and emphasizes the importance of interdisciplinary attention for patients suffering from mpox. Special emphasis should be placed on individuals support and follow-up, focusing on detecting concomitant STIs.

**Disclosures:**

**All Authors**: No reported disclosures

